# Omics Approaches in Invasion Biology: Understanding Mechanisms and Impacts on Ecological Health

**DOI:** 10.3390/plants12091860

**Published:** 2023-04-30

**Authors:** Shanshan Qi, Jiahao Wang, Yi Zhang, Misbah Naz, Muhammad Rahil Afzal, Daolin Du, Zhicong Dai

**Affiliations:** 1 School of Emergency Management, Jiangsu University, Zhenjiang 212013, China; qishanshan1986120@163.com; 2 Key Laboratory of Modern Agricultural Equipment and Technology, Ministry of Education, School of Agricultural Engineering, Jiangsu University, Zhenjiang 212013, China; 3 Institute of Environment and Ecology, School of the Environment and Safety Engineering, Jiangsu University, 301 Xuefu Road, Zhenjiang 212013, China; 4 Jiangsu Collaborative Innovation Center of Technology and Material of Water Treatment, Suzhou University of Science and Technology, Suzhou 215009, China

**Keywords:** omics invasion biology, microbe ecological health, plant–microbe interactions, microbial diversity, environmental stress

## Abstract

Invasive species and rapid climate change are affecting the control of new plant diseases and epidemics. To effectively manage these diseases under changing environmental conditions, a better understanding of pathophysiology with holistic approach is needed. Multiomics approaches can help us to understand the relationship between plants and microbes and construct predictive models for how they respond to environmental stresses. The application of omics methods enables the simultaneous analysis of plant hosts, soil, and microbiota, providing insights into their intricate relationships and the mechanisms underlying plant–microbe interactions. This can help in the development of novel strategies for enhancing plant health and improving soil ecosystem functions. The review proposes the use of omics methods to study the relationship between plant hosts, soil, and microbiota, with the aim of developing a new technique to regulate soil health. This approach can provide a comprehensive understanding of the mechanisms underlying plant–microbe interactions and contribute to the development of effective strategies for managing plant diseases and improving soil ecosystem functions. In conclusion, omics technologies offer an innovative and holistic approach to understanding plant–microbe interactions and their response to changing environmental conditions.

## 1. Introduction

**Figure 1:**
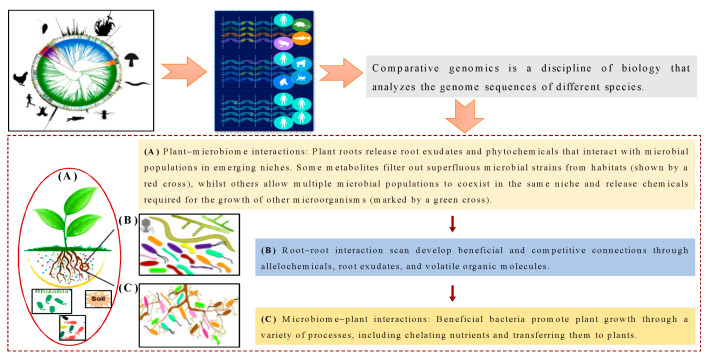
The study of plant–microbe interactions is crucial for understanding the ecological impact of invasive plant species and for developing effective management strategies.

The relationship between plant hosts, soil, and microbiota is complex and critical for maintaining healthy ecosystems and sustainable agriculture. With the advancement of omics technologies, it has become possible to simultaneously analyze the interactions between these components at a molecular level, which can lead to enhancing plant health and improving soil ecosystem functions [1]. By examining invasive species at the genetic level, researchers can identify invasive genes, expression and regulation patterns, and invasiveness genotypes, which can help dissect plant invasion and trace its evolution [2]. Omics approaches can also shed light on the relationship between soil, microbiomes, and soil health, which is crucial for maintaining terrestrial ecosystems and food security [3]. Metagenomics and transcriptomics are two commonly used omics methods in host-microbiome research. Metagenomics involves the analysis of genetic material from microbial communities in their natural environment. This approach provides a comprehensive view of the microbial diversity and functional potential of a community. Transcriptomics, on the other hand, involves the analysis of gene expression patterns in a particular organism or community [4]. This approach provides information on the active genes and functional processes at a specific time and under specific conditions. In host–microbiome research, metagenomics can be used to identify the microbial species present in a particular host and their functional potential, while transcriptomics can provide insights into the interactions between the host and its microbiota and the molecular mechanisms underlying these interactions. A combination of these two methods can provide researchers with a more comprehensive understanding of the complex relationships between host and microbiome [5]. Moreover, characterizing host–microbiome interactions using holoomics can help to better understand the system-level mechanisms underlying plant invasion and its impact on ecological health [6]. These findings can inform the development of effective management approaches such as plant breeding and field practices that reduce the spread of invasive species and promote soil health. Traditional methods in ecological health studies often involve culturing microorganisms in a laboratory, which can be time-consuming, biased, and can fail to capture the full extent of the microbial diversity present in the environment. Moreover, traditional methods are often limited in their ability to identify the functional potential of microbial communities and their interactions with other components of the ecosystem [7]. In contrast, omics approaches, such as metagenomics and transcriptomics, offer a more comprehensive and unbiased analysis of microbial communities and their functions. These methods can provide insights into the complex interactions between different components of the ecosystem, such as plants and their microbiota, and the molecular mechanisms underlying these interactions. Additionally, omics approaches can provide a wealth of data that can be used to develop predictive models and inform strategies for managing ecological health [8]. Overall, the use of omics approaches represents a significant improvement over traditional methods in ecological health research. Hence, omics approaches have the potential to significantly advance our understanding of invasion biology and its implications for ecological health. Recent technological improvements have allowed a more comprehensive approach to plant disease ecology, determining etiology and the underlying causes [9,10]. Omics tools can examine plants and microorganisms’ genotype–phenotype spectrum (Figure 1). The use of omics approaches, including genomics, metagenomics, transcriptomics, and proteomics, can help in characterizing host–microbiome interactions and identifying the functional links between plants and their associated microbes [11]. These approaches can reveal gene loci and pathways that affect colonization and community composition and help predict host fitness and control interaction outcomes. The use of holo-omics, which combines multiple omics datasets, can provide a system-level understanding of host–microbiome interactions [12]. However, the use of these techniques also requires careful experimental design and validation to ensure that the results are accurate and reproducible. Omics approaches have become increasingly important in invasion biology, as they allow researchers to gain a deeper understanding of the mechanisms and impacts of invasive species on ecological health [13]. For example, transcriptomic studies can reveal the genetic pathways that are activated in invasive species, while proteomic studies can identify the proteins that are involved in invasion processes. Metabolomic studies can reveal the biochemical pathways that are altered in invasive species, while genomic studies can provide insights into the genetic basis of invasive traits [14]. One of the key advantages of omics approaches is that they allow for studying the complex interactions between invasive species and their environment [15]. For example, a recent study used metabolomic and transcriptomic approaches to investigate the impact of an invasive plant species on the soil microbial community. The study found that the invasive species altered the soil metabolome and transcriptome, which in turn affected the composition and function of the soil microbial community [16]. Another important application of omics approaches in invasion biology is the identification of potential targets for invasive species management. For example, a recent study used transcriptomic and proteomic approaches to identify potential targets for the control of an invasive ant species [17]. The study identified several genes and proteins that are involved in the invasion process and suggested that these could be targeted for the development of new control strategies [18]. Understanding these traits can aid in discovering the mechanisms that govern plant defense ecology and developing more efficient management strategies, ranging from plant breeding to field practices that minimize disease transmission [19]. Recent research suggests that a plant’s microbiome is influenced by the host’s genetics. However, pinpointing genetic loci that impact microbial selection is challenging [20]. The genome-wide association study (GWAS) method is a potent genetic tool that allows for the identification of genetic variations linked to a specific trait or phenotype [21]. In the context of invasion biology and microbiome research, GWAS can be used to unveil microorganisms that are affected by the host genotype and gene loci that influence colonization [22]. Through a comprehensive analysis of the genetic variability across the host plant’s entire genome, scientists can pinpoint specific genes or genomic regions associated with certain microbiome compositions or the host plant’s capacity to host particular microorganisms [23]. Conventional methods for identifying microorganisms involve growing them in a laboratory, which limits their ability to recognize the diversity and functions of microbes. In contrast, omic techniques such as metagenomics and transcriptomics offer a more comprehensive and impartial view of microbial communities in their natural environment. They can identify rare or unculturable organisms and provide insights into molecular mechanisms [24]. Consequently, omic techniques inform strategies for managing ecological health and represent a considerable advancement over traditional methods.

Indeed, omics approaches are proving to be powerful tools for studying invasive species and their impacts on ecological health. By combining these techniques with traditional ecological methods, researchers can gain a more complete understanding of invasion biology, including the underlying molecular mechanisms, and develop more effective strategies for managing invasive species [25]. The impacts of invasive species, including their interactions with other components of the ecosystem, can inform decision-making to promote ecological health [26]. Implementing omics techniques is crucial for advancing our understanding of ecological health and the relationships between plant hosts, soil, and microbiota. The continued advancement of omics technologies holds great promise for providing new insights into ecosystem functions and identifying effective strategies for managing environmental threats [4]. By incorporating omics approaches into environmental management practices, we can make more informed decisions and better protect our planet’s ecological health for future generations.

## 2. Genomics as a Key Tool for Understanding Plant Invasiveness

Plant invasions have become a significant problem worldwide, resulting in the displacement of native plant species and causing ecological, social, and economic problems [27]. High-throughput genomics technologies have enabled researchers to identify key genes and pathways that are involved in plant invasiveness. In the study of Gladman et al. [28] discussed the recent advances in genomics research on invasive plants, highlighting the key insights gained into the mechanisms underlying plant invasiveness and the potential for using genomics to manage invasive species [29]. By comparing invasive plant species to non-invasive plants, researchers can identify genes and pathways that may be associated with invasive traits. This information can help in predicting and controlling invasiveness [30]. For instance, a study on the invasive plant species Japanese knotweed identified genes associated with stress response and reproduction as being crucial for its invasiveness [4]. Similarly, a study on the invasive grass species *Phalaris arundinacea* revealed genetic differences between the invasive and non-invasive populations, with genes related to stress response and growth regulation being overrepresented in the invasive populations [31]. Another important aspect of invasive plant genomics is understanding how invasive plants adapt to new environments. Research has shown that invasive plants often exhibit high levels of phenotypic plasticity, meaning they can alter their physical and physiological characteristics in response to environmental cues [32]. This plasticity may be due to genetic changes that allow invasive plants to quickly adapt to new environments. For example, a study on the invasive plant *Solidago gigantea* found that the invasive populations had genetic variations that were linked to increased plasticity and adaptation to novel environments [32,33]. Invasive plant genomics provides a powerful tool for understanding the molecular basis of plant invasiveness and can aid in predicting and controlling invasive species, as shown in Figure 1. However, it is important to also consider the ecological and evolutionary factors that contribute to invasiveness, as well as the potential impacts of management strategies on non-target species and ecosystems [34]. 

The importance of studying plant–microbe interactions lies in the context of invasive plant species. According to Figure 1, invasive plants can change the makeup and variety of soil microbial communities, which results in alterations to ecological functions.

### 2.1. Population Genomics and Their Research Method 

Population genomics is a field that examines genetic variation in natural populations at the genome level. It involves analyzing large sets of genetic data from many individuals within a population to understand patterns of variation and how they relate to various ecological and evolutionary processes [35]. In the context of plant invasions, population genomics can be used to investigate the genetic basis of invasiveness, such as identifying genetic traits that enable invasive species to thrive in new environments. This information can then be used to develop more effective management strategies for controlling invasive species [33]. One common research method in population genomics is the use of next-generation sequencing (NGS) techniques. NGS allows for the high-throughput sequencing of multiple individuals at once, generating large amounts of genetic data. This data can be analyzed using various bioinformatic tools to identify patterns of genetic variation within and between populations, as well as to detect signals of natural selection and other evolutionary processes [36]. Other methods used in population genomics include genotyping-by-sequencing (GBS), restriction-site-associated DNA sequencing (RAD-seq), and whole-genome sequencing (WGS) [37]. Each method has its own advantages and disadvantages, and the selection of a particular method depends on the research question and the species being studied. Population genomics is a powerful tool for understanding the genetic basis of plant invasiveness and can help inform management strategies for controlling invasive species [32,37,38]. Variation in gene expression affects plant development, adaptability, invasiveness, habitat circumstances, and other biological features [39]. Genes are involved in secondary metabolism, non-biological stimuli response, and development in plant rhizomes. Forward ecology and reverse genetics are similar, but the former emphasizes environmental and genetic population influences [40,41].

#### 2.1.1. Comparative Genomics 

Comparative genomics can also reveal differences in the sequences of genes involved in important biological processes, such as growth, reproduction, and stress response [42]. For example, invasive species may have allelic variants of genes that confer increased resistance to herbivores or pathogens or that allow for more efficient nutrient uptake [43]. One aspect that can vary between plant genomes is their size, structure, and sequence properties. Genome size, or the amount of DNA contained within a cell nucleus, can have various effects on plant biology, including cell size, growth rate, and adaptation to environmental conditions [44,45,46]. Large genomes can pose a risk of extinction for species; however, for invasive plants, a low nuclear DNA content and short generation time can facilitate their reproductive success and expansion, thus increasing their invasion potential [47].

**Figure 2:**
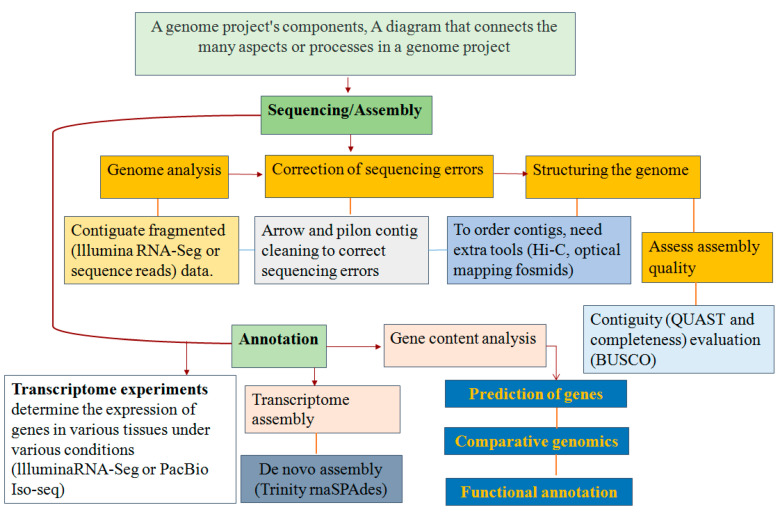
Invasion biology genome projects and elements of a genome project. A flow chart connecting the elements or steps in a genome project.

Future research in genomics to understand plant invasiveness is likely to focus on several areas. First, there is a need for more comprehensive and detailed genomic data on invasive plant species, including their functional genomics and epigenetics. This will help to identify the genes and molecular pathways that underlie invasive behavior, as well as potential targets for controlling invasiveness [48]. Second, there is a need for a better understanding of the role of the environment in shaping the genomic and epigenetic characteristics of invasive plants. This will require more integrative studies that combine genomics, transcriptomics, proteomics, and metabolomics with environmental data to identify the environmental drivers of invasive behavior and how these interact with genetic factors [49]. Third, comparative genomics across related invasive and non-invasive species can provide insights into the evolution of invasiveness and the mechanisms that drive it. This can help to identify the genetic changes that have occurred during the transition from a non-invasive to an invasive species and how these changes have contributed to the invasive phenotype [50]. Finally, there is a need for more translational research to develop practical applications for controlling invasive plants based on genomics. This may include the development of gene editing techniques for targeted gene knockouts or editing, the identification of molecular markers for screening for invasive potential, and the development of new biocontrol agents based on genomics [51] (Figure 2).

#### 2.1.2. Role of the Soil Microbiome 

The soil microbiome can play a key role in plant invasion biology. The soil microbiome is a complex community of microorganisms that interact with each other and with plants in the soil environment [52]. These microorganisms can have positive, negative, or neutral effects on plant growth and health, and can therefore have a significant influence on plant invasions [53]. For example, some invasive plant species have been found to have a different microbiome than native plant species, which may give them a competitive advantage in certain environments. Additionally, changes in the soil microbiome due to factors such as land use change, climate change, or the introduction of invasive species can alter plant–microbiome interactions and affect the success of invasive plants [52]. However, more studies are needed to fully understand the complex interactions between invasive plants and the soil microbiome and to develop effective management strategies [54,55]. Therefore, studying the soil microbiome and its interactions with plants can provide important insights into plant invasion biology and develop strategies for invasive species management.

#### 2.1.3. Impacts of Plant Invasion on Soil Ecological Functions 

The invasion of plant species can cause substantial effects on soil ecological functions, such as nutrient cycling, carbon sequestration, and soil structure [56]. The invasion of non-native plant species can modify the diversity and composition of soil microbial communities, resulting in alterations in nutrient cycling and soil organic matter dynamics [57]. Additionally, invasive plants can diminish soil aggregation and water infiltration, potentially leading to a reduction in soil fertility and erosion [58]. Genomic technologies can help us to better understand how non-native plant species affect soil ecological functions. For example, metagenomic sequencing can identify changes in microbial community composition and function in invaded soils [59]. Transcriptomics enables the identification of upregulated or downregulated microbial genes in response to invasive plants, which can explain the mechanisms responsible for such changes [60]. The technique of stable isotope probing can help to identify the microbial groups that carry out specific functions in the soil, as well as how these functions are impacted by the plant invasion [61]. Overall, soil microbes can play a crucial role in maintaining soil health and successful plant invasions [3].

## 3. The Multi-Omics Methods for Plant Invasion 

The use of multi-omics methods has gained popularity in the field of plant invasion biology because of their capacity to offer a broad comprehension of the molecular mechanisms in invasive plant species and their impact on ecological health [62,63]. Multi-omics methods are capable of identifying potential targets for invasive species management. For example, a study of the invasive plant species *Centaurea stoebe* found that genes involved in phenylpropanoid biosynthesis, a process that produces compounds that may have allelopathic effects on native plant species, were upregulated in the invasive population. These findings suggested that targeting the phenylpropanoid biosynthesis pathway may be an effective strategy for controlling *C. stoebe* [64]. In addition to identifying the molecular mechanisms underlying invasiveness, multi-omics methods can also reveal the ecological impacts of invasive species. A study of the invasive tree species *Ailanthus altissima* found that its invasion was associated with changes in soil microbial communities and nutrient cycling. These findings suggested that *A. altissima* may have a significant impact on ecosystem functioning beyond its direct competitive interactions with native plant species [65]. Multi-omics approaches can aid in the development of effective management strategies for invasive species and contribute to the sustainability of biodiversity and ecosystem functioning by offering a comprehensive view of the molecular mechanisms that underlie invasiveness and the ecological impacts of invasive species. 

Omics technologies, including transcriptomics, proteomics, metabolomics/metabonomics, genomics, microbiomics, and nutrigenomics, have revolutionized the study of invasion biology and ecology. By utilizing these technologies, scientists are capable of analyzing the genetic, metabolic, and ecological mechanisms that contribute to the invasion of non-native species, as well as the consequences of these invasions on native ecosystems [65]. In this discussion, we explore some of the crucial uses of omics in the fields of invasion biology and ecology.

*Transcriptomics:* Transcriptomics is a valuable approach in invasion biology for detecting genes and pathways that play a role in invasion success, including those linked to stress tolerance, nutrient acquisition, and growth rate [66,67,68]. Furthermore, transcriptomics can uncover genes associated with the interplay between invasive and native species, including the production of allelopathic compounds and other signaling molecule. However, study, Qi et al. [69] reported that a significant number of genes, including candidate genes, were linked to plant-pathogen interactions and stress response in *S. trilobata*. Several recognition, signaling, and defense genes were differentially regulated among treatments, as validated by qRT-PCR. These findings highlight the genes and molecular associations responsible for plant defense against a rapidly proliferating invasive clonal weed, and they can serve as a valuable resource for future research on disease resistance mechanisms and invasive plant management. The research conducted by Zhang et al. [70] revealed that certain transcription factors linked to plant stress, including APETALA2/ethylene response factors, exhibited up-regulation, while others such as zinc-finger proteins experienced down-regulation. Moreover, the allelochemicals present in *C. canadensis* induced the up-regulation of detoxification genes (DTX), genes associated with reactive oxygen species (ROS), calcineurin B-like interacting protein kinases (CIPKs), and calmodulin, offering novel insights into the molecular-level allelopathy in *C. canadensis* and advancing the understanding of the invasion mechanisms employed by non-native plant species. Luo et al. [71] reported that changes in histones may play a role in the divergent expression of cold-responsive genes between the two populations, potentially enabling them to better respond to chilling stimuli and adapt to their respective environments. To comprehensively examine the cold tolerance of alligator weed, transcriptomics analysis uses high-throughput sequencing technology to assess gene expression under cold stress conditions. This method enables the identification of differentially expressed genes, pathways, and regulatory networks that contribute to alligator weed’s response to cold stress. Furthermore, a study by Saminathan et al. [72] revealed that the common gene expression patterns for different pathways in two plant systems that grow in mine sites with toxic waste suggest that both invasive plants had developed mechanisms to adapt to and survive in these harsh environments. The fact that both plant systems had few common heavy-metal pathway regulators addressing mineral toxicity/deficiency further supports this idea and suggests that these invasive plants are able to efficiently utilize available resources for growth and development.

*Proteomics:* Proteomics is a powerful tool for studying the proteins expressed by an organism or a system. In the context of invasive plants, proteomics can provide insights into the molecular mechanisms underlying their success in new environments, as well as the interactions between the plants and their associated microbial communities [73]. For example, a proteomics study of the invasive plant species *Spartina alterniflora* found that it produces proteins that enable it to tolerate high levels of salt and other environmental stressors, which may contribute to its ability to outcompete native plant species in coastal ecosystems [74]. Another study of the invasive plant species *Acacia longifolia* identified proteins involved in nutrient acquisition and transport, suggesting that the plant is able to efficiently extract and utilize resources from its new environment [75]. Proteomics can also be used to study the interactions between invasive plants and their associated microbial communities. For example, a study of the invasive plant species *Ageratina adenophora* found that it produces proteins that interact with the microbial communities in its rhizosphere, potentially influencing nutrient cycling and other ecosystem processes [76]. Furthermore, a study on the European invasive species Asteraceae Solidago canadensis, which had colonized the soil of a former cokery, exhibited a distinct pattern in its leaf proteome and physiological response, as shown in (Table 1). In general, proteomics serves as a valuable tool to explore the molecular mechanisms that drive plant invasions and can assist in devising management strategies for invasive species and the restoration of ecosystems.

*Metabonomics*: Metabonomics involves the analysis of small-molecule metabolites in organisms [14,77]. In invasion biology, metabonomics can be used to identify the metabolites involved in invasion success, such as those involved in nutrient acquisition, detoxification, and stress tolerance [78,79,80]. Metabonomics can also be used to identify changes in the metabolome of native species in response to invasive species, providing insights into the impacts of invasion on ecosystem function [4,81]. A study focusing on the metabolic profiling of soil samples from invaded and non-invaded areas revealed that invasive plants can alter soil microbial community compositions and nutrient cycling, leading to changes in soil metabolites [3]. However, Xiong et al. [82] reported the use of metabonomics to identify changes in the metabolic profiles of plants and soil microbes in response to invasion by a non-native plant species, revealing the metabolic pathways involved in plant–microbe interactions. Yin et al. [53] applied metabonomics to analyze the metabolites of soil samples from a plant–fungi–nematode complex, revealing the metabolic and functional roles of the rhizosphere bacteria in this system. Furthermore, van der Heijden et al. [83] explained the metabolic pathways involved in the association between mycorrhizal fungi and plants, revealing the important role of these fungi in nutrient cycling and plant growth. Another study by Mhlongo et al. [84] used metabonomics to investigate the effects of arbuscular mycorrhizal fungi on the metabolic profiles of plants and soil microbes, revealing the metabolic pathways involved in the plant–microbe interactions.

*Microbiomics*: Microbiomics involves the analysis of microbial communities in ecosystems. In invasion biology, microbiomics can be used to study the interactions between invasive and native microbial communities [54,85], as well as the impacts of these interactions on ecosystem function [52,86]. Microbiomics can also be used to identify potential targets for the microbial control of invasive species [52]. According to Zhang et al. [87], an increase in invasion levels by *C. canadensis* had a notable impact on the structure of soil microbiota, causing significant changes in the relative abundance of various bacterial and fungal taxa, some of which were critical for nutrient cycling. The changes in soil’s biotic and abiotic composition caused by *C. canadensis* invasion may trigger positive plant–soil feedback mechanisms that could facilitate the establishment and expansion of this invasive weed. According to Mei et al. [88], the functional traits that contribute to the invasiveness of the clonal plant *W. trilobata* are related to both its environmental adaptability and the endophytic bacterial community. These findings suggest that such functional traits may promote the plant’s invasiveness, thereby increasing its likelihood of success in invading new habitats.

*Nutrigenomics*: Plant nutriomics is a field within nutrigenomics that combines genetics, molecular biology, and bioinformatics to understand the complex interactions between plants, soil, and microbes in relation to plant nutrient acquisition, utilization, and metabolism [89]. The goal of plant nutriomics is to develop more efficient and sustainable agricultural practices by identifying the genetic and molecular mechanisms underlying plant responses to environmental stress and nutrient deficiencies [90]. One of the main objectives of plant nutriomics is to improve plant nutrient use efficiency (NUE), which is the ability of a plant to take up and utilize nutrients from the soil. This is particularly important in the context of population growth and environmental restrictions, where there is a growing demand for food production while also ensuring the sustainable use of resources [91]. By enhancing NUE, plant nutriomics can increase agricultural productivity, reduce the need for fertilizers, and mitigate the negative environmental impacts associated with fertilizer overuse. Plant nutriomics involves the use of various omics techniques, including genomics, transcriptomics, proteomics, and metabolomics, to study the molecular mechanisms involved in plant–nutrient–microbe interactions [92]. For example, transcriptomic analysis can be used to identify genes that are differentially expressed in response to nutrient deficiency or stress [93]. Metabolomic analysis can be used to identify the key metabolites involved in nutrient acquisition and utilization. The information generated from plant nutriomics research can be used to develop new plant breeding strategies, such as marker-assisted selection, for improving NUE in crops [94]. Additionally, the identification of the key genes and metabolites involved in nutrient acquisition and utilization can lead to the development of new fertilizers and soil amendments that can enhance plant growth and productivity [95]. Omics technology can also provide insight into the long-term ecological effects of invasions and aid in the development of restoration plans that consider internal and external patch memory. Finally, omics technology can help in preserving ecological memory by providing a baseline understanding of native species’ genetics and biochemical mechanisms, which can be used as a reference for future ecosystem changes.

**Table 1:** Proteomics study in invasive plants, there are very few proteomics studies that have specifically focused on invasive plants.

Invasive Plant Species	Techniques of Omic	Findings of Study	References
*Ageratina adenophora* (crofton weed).	Proteomic (root exudates)	Identified proteins involved in allelopathy, which may contribute to the invasiveness of the plant.	[96,97,98]
*Acacia saligna* (golden wattle).	Proteomics (N fixing root nodules)	Identified protein elaboration in N fixation and transport; it enhances the plant’s growth and competitive ability in nutrient-poor soils.	[99]
*Microstegium vimineum* (Japanese stiltgrass).	Proteomic (invasive and native populations)	Identified differences in protein expression related to photosynthesis, stress response.	[73]
*Cytisus scoparius* (Scotch broom).	Proteomics (leaves and roots)	Identified proteins involved in plant defense, nutrient uptake.	[100]
*S. alterniflora.*	Chemico-proteomics	The function of H2S signaling in the adaptation of an invasive plant species and the important role of H2S adaptation in *S. alterniflora* to saline environments.	[101]
*R. solanacearum.*	Proteomics	Plant–bacterium interactions.	[102]
Incompatible rice/*Magnaporthe grisae*.	Proteomics	Plant–pathogen relationship; it is important in apoplastic protein patterns that occur during pathogen infection.	[103]
Potato with *Ralstonia solanacearum* UW551.	Proteomics	T3Es of *R. solanacearum* can subvert potato root immune-related proteins in a redundant manner.	[104]
*Tomato (Solanum lycopersicum) fruit was invaded by Sclerotinia rolfsii.*	Proteomics	To prioritize candidate proteins for storage organ quality improvement.	[105]
*Aspergillus terreus* invades *Solanum tuberosum* L.	Proteomics	During colonization, *TA. terreus* differently activated enzymes in potato tubers.	[106]
Phytophthora infestans, the pathogen responsible for potato late blight.	Proteomics	The potential magnitude of proteins encoded in the genome.	[107]
Expressed in *Nicotiana benthamiana*, *R. solan.*	Proteomics	Pathogens can adapt to the specific host they encounter.	[108]
Interactions between plants and viruses, bacteria, fungi, and nematode.	Proteomics	Interactions between plants and viruses, bacteria, fungi, and nematodes were identified and reported in proteomic studies.	[109]
*Arabidopsis thaliana* plants.	Proteomics	Providing insight into the signaling networks of a particular cell type.	[110]
The symbiotic interaction between *Brassica napus* and *Piriformospora indica*.	Proteomics	GO and KEGG pathway analysis revealed gene sets involved in metabolic processes.	[111]
*Magnaporthe oryzae* (*M. oryzae*).	Proteomics	Response to *M. oryzae* invasion; the iTRAQ approach was utilized to identify differentially expressed proteins (DEPs) in both the durable, resistant rice variety Gangyuan8 (GY8) and the susceptible rice variety Lijiangxintuanheigu (LTH).	[112]
Study interactions between plants and pathogens	Proteomics	Interactions between plants and pathogens in compatible systems.	[113]
Potato, a model for periderm.	Proteomics	Early tuber growth in potatoes; periderm tissue replaces the epidermis.	[114]
Microbial pathogens.	Proteomics	Bacterial interactions among distinct bacterial taxa, including symbiotic, pathogenic, and commensal bacteria.	[115]
Tomato	Proteomics	Proteome study investigation of the dynamics of various disease responses in tomato.	[116]
Hybrids of *Solanum* differing in resistance to *Dickeya solani.*	Proteomics	Significant differences were observed in the large-fold of various proteins between resistant and susceptible potato cultivars, and diploid clones were induced.	[117]
Proteomics toward the improvement of crop productivity and stress resistance.	Proteomics	The limitations of non-model organism proteomics techniques and data interpretation.	[118]
Plant.	Proteomics	Plant-specific issues on how proteomics can help plant systems biology.	[119]
Plant.	Proteomics	Plant proteomics is currently in its early stages and is subject to a significant impact on plant biology.	[120]
*Alternanthera philoxeroides* (*Alligator* weed).	Proteomics	The response of *Alternanthera philoxeroides* roots stems, and leaves to potassium deficiency stress.	[121]
Gibberella stalk rot in maize.	Proteomics	The defense response of corn stalks against graminearum, proteins from various immune-related pathways.	[122]
Rice in biotic stress.	Proteomics, metabolomics	Proteins and metabolites defense response of rice to biotic stress.	[123]

As mentioned earlier, there are very few proteomics studies that have specifically focused on invasive plant species and that have suggested that invasive plants may have unique proteomic profiles compared to native species, which may contribute to their invasive potential.

For example, a study by Castro-Díez et al. [124] compared the proteomic profiles of two closely related invasive plant species, *Acacia longifolia* and *A. melanoxylon*, with those of their native congeners. The study found that the two invasive species had higher levels of proteins involved in stress response and defense, as well as proteins involved in photosynthesis and energy production, compared to the native species. These findings suggested that the invasive species may have evolved unique proteomic adaptations to cope with the stresses associated with invasion, such as nutrient-poor soils, competition, and herbivory. Another study by Li et al. [125] compared the proteomic profiles of invasive and native populations of the plant species *Solidago canadensis* (*Canada goldenrod*). The study found that the invasive populations had higher levels of proteins involved in photosynthesis, stress response, and defense compared to the native populations. These findings suggest that the invasive populations may have adapted to their new environment by increasing their capacity for energy production and stress tolerance. Overall, these studies (Table 1) suggest that proteomics can be a useful tool for understanding the molecular mechanisms underlying plant invasion and may help to identify potential targets for the management and control of invasive species. However, more research is needed to fully understand the proteomic adaptations of invasive plants and their role in the invasion process.

## 4. Invasive Species and Environmental Change 

Invasive species can have significant impacts on environmental change, management, and health, and effective management strategies are necessary to prevent and control their spread, which include early detection, rapid response, and the prevention of new invasions. Biosecurity measures, such as inspections at borders, can inhibit the effects of invasive species. Additionally, monitoring and control programs can be implemented to prevent the spread and founding of invasive species in new spaces [126], as they can cause significant damage to crops, livestock, and other natural resources, leading to losses in agricultural and forestry industries. Invasive species can also cause significant economic losses in tourism and recreation industries, as they can negatively affect the aesthetic and recreational value of ecosystems. Furthermore, invasive species can have negative impacts on human health, as they can be vectors for diseases or produce toxic substances. Managing invasive species requires a coordinated effort between governments, organizations, and individuals to prevent their effects, perceive and react to novel invasions, and control and eradicate established populations. This may involve a range of strategies, such as developing early warning systems, implementing quarantine measures, conducting research to understand the ecology of invasive species, and using integrated pest management approaches to control established populations [81]. Management strategies are essential to controlling the spread of invasive species and mitigating the effects of environmental change on human health [82]. These strategies include measures such as early detection and rapid response, habitat restoration, and public education campaigns. By working to manage these issues related to invasion ecology effects, we can help protect both the environment and human health [81].

### Review of the Application of Omics to Invasion Biology and Ecology

Omics approaches can provide a comprehensive view of the molecular mechanisms underlying invasive species’ success and their impact on native ecosystems [49]. Future research in this area should focus on integrating omics approaches with ecological studies to gain a better understanding of the complex interactions between invasive species and their environment. Moreover, the development of new technologies for the analysis of complex omics data will allow for more efficient and effective studies of invasive species [50]. 

The connection between climate change and biological invasion has been discussed, focusing on how climate change can increase habitat disturbance and the prevalence of invasive species. The effects of climate change on biological invasion can be studied using genetic and genomic technologies [51], such as meta-barcoding and meta-genomics, which can analyze the whole genome of invading organisms to detect fast-changing regions and genetic contamination. Genomic markers can be used to monitor the origins of invading organisms more reliably and measure community effects [52]. To enhance understanding of the complex relationships between invasive species and their environment, future studies in this field should focus on integrating omics techniques with ecological research. Additionally, the development of new technologies for analyzing complex omics data will enable more efficient and streamlined analysis of invasive species [53].

## 5. Conclusions and Remarks

The generation mechanisms of omics information have improved in recent years. New technologies such as third-generation sequencing can improve researchers’ data. These developments have helped scientists to combine omics data. This will help in describing microbial functions and understanding their role in complex ecosystems. This study evaluated the high-dimensional system-level strategies for simultaneously analyzing organisms and systems. In recent times, advancements in omics technologies have significantly enhanced our ability to generate high-quality molecular data, enabling us to better comprehend the functions of microorganisms and their roles in complex ecosystems. Third-generation sequencing has further augmented the quantity and quality of omics data accessible to researchers, facilitating a more comprehensive and integrated analysis of microbial systems.

This review specifically highlighted the development and utilization of high-dimensional system-level approaches to analyze organisms and systems concurrently, which can provide a more complete understanding of the interactions between microorganisms and their environment. These strategies hold the potential to uncover crucial insights into microbial function and evolution and could lead to groundbreaking discoveries in fields such as biotechnology, medicine, and environmental science. The emphasis of this review on the importance of molecular data and fitness function comparison between native and introduced ranges is also commendable. It is true that the environment plays a significant role in shaping phenotypic and epigenetic alterations in invasive plant species. By examining these variations, we can gain a better understanding of the mechanisms driving the invasiveness of these species and develop effective management strategies to control their spread. Combining parallel studies is also a valuable approach, as it can allow us to integrate data from multiple sources and perspectives to obtain a more holistic understanding of the invasion process. Generally, this review provided valuable insights into the study of the molecular adaptability of invasive plants and highlights important avenues for future research.

## Data Availability

There is no data availability statement.
